# Editorial: Insights in bone research 2021

**DOI:** 10.3389/fendo.2022.979276

**Published:** 2022-07-26

**Authors:** Jonathan H. Tobias, Giacomina Brunetti

**Affiliations:** ^1^ Muscoloskeletal Research Unit, Translational Health Sciences, and Medical Research Council (MRC) Integrative Epidemiology Unit, Population Health Sciences, Bristol Medical School, University of Bristol, Bristol, United Kingdom; ^2^ Department of Biosciences, Biotechnologies and Biopharmaceutics, University of Bari Aldo Moro, Bari, Italy

**Keywords:** Bone mineral density, Rickets and osteomalacia, diabetes, Obesity, guideline, X-linked hypophosphatasia, osteoarthritis, bone

This Research Topic comprises papers related to rare bone diseases, osteoporosis and osteoarthritis, illustrating the wide spectrum covered by the Bone Research section of Frontiers in Endocrinology.

Laurent et al. present consensus recommendations for the diagnosis and Management of X-linked hypophosphatemia (XLH) in Belgium. In this disease, *PHEX* mutations determined the increase of the levels of the hormone fibroblast growth factor 23 (FGF23), with consequent renal phosphate wasting, altered skeletal and dental mineralization. Though evidence-based guidelines in this area have previously been published, there is a need to translate the principles on which these are based to more practical recommendations at a national level, in this instance a relatively well funded mixed state and private healthcare system. The broad but nevertheless detailed and practical overview of the clinical aspects of diagnosis and management of XLH presented in this article provides useful guidance for management of these patients. Guidance on the use of burosumab in children with XLH is particularly timely, given the recent EMA market authorization of this new FGF23 antibody treatment, which has the potential to transform the lives of those living with this condition.

Two articles report findings from clinical studies aiming to examine the basis for increased fracture risk in diabetes, by studying mechanisms contributing to cross talk between bone and energy metabolism. Starup-Linde et al. examined the role of osteoglycin in the altered bone turnover associated to type 1 and type 2 diabetes (T1D and T2D, respectively), based on studies in mice suggesting that osteoglycin exerts endocrine effects on bone and pancreas. Osteoglycin is a proteoglycan derived from bone, cartilage and myocytes; it enhances bone formation and mineralization. The authors did not find significant differences for osteoglycin levels between T1D and T2D patients, but interestingly they showed that osteoglycin levels in diabetic patients were unrelated to metabolic markers of diabetes and bone turnover markers, arguing against such a role. A related study by Cherian et al. examined whether two adipomyokines, irisin and Meteorin-like (METRNL), play a role in altered bone remodeling in individuals with obesity and T2D. Irisin and Meteorin-like (METRNL) contribute to increased thermogenesis following exercise and cold exposure, and affect bone cell activity. Levels of irisin and METRNL were found to be related to several molecules with osteogenic properties, suggesting that adipomyokines might affect the development of bone and muscle complications associated with obesity and T2D.

The study by Samvelyan et al. used a murine model to examine the pathogenesis of osteoarthritis. Specifically, the authors examined whether altered growth plate dynamics contribute to the development of osteoarthritis in the destabilisation of medial meniscus (DMM) model, as well as in a non-surgical loading model. In both models, development of osteoarthritis was associated with switching of articular chondrocytes to a hypertrophic chondrocyte phenotype, associated with morphological changes including growth plate bridging. Consistently, the authors demonstrated the increased expression of hypertrophy markers Col10a1 and MMP13 in the growth plate of osteoarthritis models. Additionally, epiphyseal trabecular bone abnormalities were associated to morphological changes of the growth plate. Thus, these results further support the role that the altered growth plate with the consequent bone disease may play in the development of osteoarthritis.

Taken together, these four papers exemplify the range of insights in the bone field in 2021.

## Author contributions

All authors listed have made a substantial, direct, and intellectual contribution to the work and approved it for publication.

## Conflict of interest

The authors declare that the research was conducted in the absence of any commercial or financial relationships that could be construed as a potential conflict of interest.

## Publisher’s note

All claims expressed in this article are solely those of the authors and do not necessarily represent those of their affiliated organizations, or those of the publisher, the editors and the reviewers. Any product that may be evaluated in this article, or claim that may be made by its manufacturer, is not guaranteed or endorsed by the publisher.

